# Effects of specimen haemolysis on complete blood count results by Abbott Alinity hq System

**DOI:** 10.11613/BM.2021.030706

**Published:** 2021-10-15

**Authors:** Müjgan Ercan, Emiş Deniz Akbulut, Nihayet Bayraktar, Şerif Ercan

**Affiliations:** 1 Department of Biochemistry, Afyonkarahisar Health Sciences University, Afyonkarahisar, Turkey; 2 Department of Biochemistry, Ankara City Hospital, Ankara, Turkey; 3 Department of Biochemistry, Faculty of Medicine, Harran University, Sanliurfa, Turkey; 4 Department of Biochemistry, Lüleburgaz State Hospital, Kırklareli, Turkey

**Keywords:** complete blood count, reference change values, haemolysis, interference

## Abstract

**Introduction:**

The current study aimed to assess the interference of *in vitro* haemolysis on complete blood count (CBC) using Abbott Alinity hq system, and to determine which haemolysis levels affect the reliability of sample results.

**Materials and methods:**

Blood samples obtained from 25 volunteers in K3-EDTA tubes were divided into four aliquots. The first aliquot was not subjected to any intervention. The second, third and fourth aliquots were passed through a fine needle 2, 4 and 6 times, respectively. Complete blood count was performed by multi-angle polarized scatter separation technology and haemolysis index (HI) was assessed from the plasma samples separated by centrifugation. Five groups were formed according to the HI values. The percentage biases between the results of non-haemolysed and haemolysed groups were compared with the desirable bias limits from The European Federation of Clinical Chemistry and Laboratory Medicine database and reference change values (RCVs).

**Results:**

In groups 1 to 4, the effects of haemolysis on CBC parameters were acceptable comparing to the analytical bias except for lymphocytes (7.26%-7.42%), MCH (2.59%), and MCHC (0.47%-2.81%). Results of group 5 (gross haemolysis) showed decreases in HCT(- 4.56%), RBC (- 4.07%) count and increase in lymphocyte (11.60%) count higher than the analytical performance specifications. Moreover, variations in MCH (4.65%) and MCHC (5.24%) were exceeding the RCVs.

**Conclusions:**

Gross haemolysis (haemoglobin concentration > 10 g/L) is likely to produce unreliable CBC results on non-pathological samples. Further studies including pathological specimens are needed.

## Introduction

Today, laboratory results are known to have considerable effect on clinical decision-making. Sample quality ensured by appropriate sampling techniques is critical in order to obtain accurate results. Errors occurring in the preanalytical phase are accepted as the main source leading to erroneous results (*1*). *In vitro* haemolysis is one of the most common preanalytical error affecting many test results. Release of haemoglobin (Hb) and the other intracellular contents secondary to impaired membrane integrity of red blood cells (RBC) may cause artifactually increase or decrease results. Inappropriate techniques for collection, transport and processing of samples are the major causes of *in vitro* haemolysis. Haemolysis may simply be detected by visual inspection of the serum or plasma component of the sample when it contains enough quantity of Hb to notice. More recently, automated analysers can quantify cell-free Hb by photometric measurement at different wavelengths following centrifugation (*2*-*9*).

However, recognition of haemolysis before analysis is unlikely and impractical for complete blood count (CBC) as whole blood specimens are used. For this reason concomitant serum or plasma samples may be evaluated for haemolysis or dramatically increased mean corpuscular haemoglobin concentration (MCHC) may be used as warning for the prevention of unnoticed haemolysis (*10*, *11*). Determining the effect of haemolysis on CBC parameters is crucial. Accordingly, defining limits to release the results safely or rejecting the sample to avoid related undesirable events may be achieved.

To date, only a few studies have investigated the effect of haemolysis on CBC parameters. The Alinity hq analyser (Abbott, Santa Clara, USA) employs optical scatter and fluorescence technologies as well as cyanide-free absorption haemoglobinometry to perform CBC (*12*). Multi angle polarized scatter separation (MAPSS) technology used in this system utilizes seven light scatter detectors and a fluorescence detector to collect a unique signal signature on each cell, without using impedance technic (*12*). To the best of our knowledge, there is no interference study related with this recently released device in the literature.

Therefore, the current study aimed to assess the interference of *in vitro* haemolysis on complete blood count using Abbott Alinity hq system, and to determine which haemolysis levels affect the reliability of sample results.

## Materials and methods

### Subjects

This cross-sectional study was conducted at Harran University Faculty of Medicine, Biochemistry Laboratory. A total of 25 volunteers were included in the study between January and March 2021. Ethical approval was taken for the study protocol from the Ethical committee of Harran University Faculty of Medicine and written informed consent was obtained from all participants (Approval Number: 14.09.2020/ HRU/20.16.10).

### Methods

Blood samples were drawn into two 3.0 mL evacuated tubes containing K3- ethylenediaminetetraacetic acid (EDTA) (Ref number:IST413) (Isotherm, Istanbul, Turkey) and mixed gently immediately after collection. Samples were portioned in order to obtain four aliquots of 1.5 mL from each subject subsequently. The first aliquot did not undergo any intervention while the second, third and fourth aliquots were passed through a small calibre needle (22 gauge, 0.70x38mm) 2, 4 and 6 times, respectively in order to produce scalar amounts of haemolysis according to the Dimeski method (*13*). Routine CBC was performed in all samples on Abbott Alinity hq System (Abbott, Santa Clara, USA) using the same batch of reagents (Abbott, Santa Clara, USA). We conducted an internal quality control process using 3 levels of Alinity h-series Control 29P (Ref. 04U7212, Abbott, Santa Clara, USA) at 20 days. The haematological parameters analysed were as follows: white blood cell count (WBC), neutrophil (neu), lymphocyte (lym), monocyte (mono), eosinophil (eos), red blood cell (RBC), haemoglobin (Hb), haematocrit (HCT), mean corpuscular volume (MCV), mean corpuscular haemoglobin (MCH), mean corpuscular haemoglobin concentration (MCHC), red cell distribution width (RDW), platelet (PLT) and mean platelet volume (MPV).

Afterwards, all aliquots were centrifuged at 1000xg for 10 minutes. Haemolysis index was measured in plasma samples on Siemens Atellica CH chemistry analyser (Siemens Healthineers, Erlangen, Germany). The HI had 7 semi-quantitative values (0 – 6) corresponding to Hb concentrations of ≤ 0.10, 0.11-1.30, 1.31-2.49, 2.50-4.99, 5.00-7.49, 7.50-9.99 and ≥ 10 g/L, respectively. All samples were grouped according to HI values.

### Statististical analysis

The Bland-Altman plot analysis is used to asses the biases between the results of paired groups. Effect of haemolysis interference was evaluated according to the analytical performance specifications (APS) for desirable bias as obtained from the European Federation of Clinical Chemistry and Laboratory Medicine (EFLM) European Biological Variation Study (*14*). Reference change values (RCVs) were accepted as clinically acceptable limit and derived from within-subject biological variation (CV_W_) and analytical variation (CV_A_) with the formula: RCV = ± 1.96 x (CV_A_^2^ + CV_W_^2^) ^½^ (*15*). Total CV_A_ was estimated from the results of internal quality control at three concentration levels. Data analyses were performed using MedCalc Statistical Software version 19.1 (MedCalc Software Ltd, Ostend, Belgium).

## Results

At the beginning, the study included a hundred portions with non-pathological results. Later, 16 portions were excluded from the study because plasma volumes obtained after CBC testing were inadequate for the assessment of HI. Twenty-five portions had no haemolysis and classified as HI 0 (Hb concentration: < 0.10 g/L, N = 25). The samples were presented as 5 groups according to the HI values; Group 1: HI 1 (Hb concentration: 0.11-1.30 g/L, N = 10), Group 2: HI 2 (Hb concentration: 1.31-2.49 g/L, N = 17), Group 3: HI 3 (Hb concentration: 2.50-4.99 g/L, N = 16), Group 4: HI 4 (Hb concentration: 5.00-7.49 g/L, N = 7), and group 5: HI 6 (Hb concentration: > 10.00 g/L, gross haemolysis, N = 9). There were no samples with HI 5 (Hb concentration 7.50–10.0 g/L). As the degree of haemolysis was different among the samples due to mechanical trauma and to determine the relative percent bias accurately HI 0 groups were paired separately for each group from the non-haemolysed portions (N = 25). Bias percentages and confidence intervals determined for each paired group are presented in Table 1.

**Table 1:** The effects of haemolysis on complete blood count

**Parameter**	**Group 1** **(N = 10)**		**Group 2** **(N = 17)**		**Group 3** **(N = 16)**		**Group 4** **(N = 7)**		**Group 5** **(N = 9)**			**APS, %**			
**Haemolysis**	**0**	**1**	**0**	**2**	**0**	**3**	**0**	**4**	**0**		**6**	**Bias**	**CV_A_**	**CV_G_**	**RCV**
WBC (x10^9^ /L)	8.01 (5.78-10.65)	7.80 (6.09-11.35)	8.01 (6.53-9.11)	7.87 (6.47-9.59)	6.90 (5.15-9.61)	7.05 (4.98-10.13)	8.01 (6.15-10.80)	7.98 (6.42-10.80)	6.16 (5.20-8.92)		6.67 (5.30-8.96)				
Bias (95%CI)	2.73 (-1.04 to 6.51)		2.04 (-0.09 to 4.17)		2.52 (0.38 to 4.66)		2.52 (-1.99 to 7.03)		4.00(1.07 to 6.93)			± 4.9	1.82	10.80	30.6
Neu (x10^9^/L)	4.71 (3.10-7.61)	4.64 (3.33-8.12)	4.71 (2.83-5.55)	4.77 (2.94-5.92)	3.41 (2.57-6.54)	3.43 (2.71-6.99)	4.44 (3.15-6.54)	4.66 (3.11-6.82)	3.46 (2.71-4.47)		3.55 (2.86-4.68)				
Bias (95%CI)	3.38 (-0.20 to 6.97)		2.83 (0.71 to 4.96)		2.37 (-1.27 to 6.01)		2.23 (-1.99 to 6.46)		4.00 (0.17 to 7.84)			± 6.9	2.49	14.00	39.42
Lym (x10^9^ /L)	2.08 (1.75-2.36)	2.20 (1.81-2.41)	2.28 (2.01-2.98)	2.27 (2.01-3.00)	2.09 (1.51-2.29)	2.24 (1.62-2.54)	2.19 (2.01-2.39)	2.33 (2.02-2.63)	1.77 (1.43-2.76)		2.17 (1.57-2.92)				
Bias (95%CI)	3.48 (-3.00 to 9.95)		2.61 (-0.73 to 5.96)		7.26 (2.84 to 11.68)		7.42 (1.45 to 9.08)		11.60 (5.57 to 17.62)			± 6.3	2.90	10.80	31.00
Mon (x10^9^ /L)	0.64 (0.45-0.68)	0.62 (0.44-0.73)	0.60 (0.50-0.68)	0.56 (0.46-0.67)	0.52 (0.44-0.67)	0.51 (0.43-0.66)	0.60 (0.51-0.68)	0.58 (0.55-0.69)	0.53 (0.41-0.57)		0.50 (0.42-0.58)				
Bias (95%CI)	1.83 (-6.08 to 9.73)		-4.27 (-11.05 to 2.49)		-1.80 (-9.1 to 5.51)		2.31 (-6.47 to 11.08)		-0.84 (-7.57 to 5.88)			± 6.5	6.85	13.30	41.47
Eos (x10^9^ /L)	0.25 (0.11-0.47)	0.26 (0.11-0.43)	0.27 (0.17-0.47)	0.25 (0.20-0.49)	0.24, (0.11-0.38)	0.24 (0.09-0.38)	0.16 (0.09-0.32)	0.14 (0.08-0.34)	0.22 (0.06-0.33)		0.18 (0.04-0.33)				
Bias (95%CI)	-2.63 (-12.49 to 7.23)		1.99 (-5.20 to 9.18)		-3.81 (-14.35 to 6.72)		-3.00 (-13.00 to 7.01)		-1.03 (-29.11 to 27.04)			± 16.8	9.48	15.00	49.19
RBC (x 10^12^/L)	4.78 (4.48-5.04)	4.90 (4.54-5.08)	4.78 (4.23-5.12)	4.87 (4.29-5.16)	4.84 (4.63-5.17)	4.85 (4.69-5.21)	4.90 (3.96-5.27)	4.90 (3.95-5.21)	4.74 (4.46-5.07)		4.50 (4.30-4.85)				
Bias (95%CI)	1.08 (0.15 to 2.01)		0.81(-0.22 to 1.84)		0.90(-1.33 to 3.12)		-1.11 (-2.74 to 0.53)		-4.07 (-5.52 to -2.62)			± 1.8	0.69	2.60	7.46
Hb (g/L)	128 (119-146)	129 (122-143)	135 (118-149)	136 (119-148)	142 (127-153)	141 (129-155)	142 (120-158)	143 (123-158)	133 (121-149)		137 (122-145)	± 1.6	0.65	2.70	7.70
Bias (95%CI)	1.23 (-0.64 to 3.11)		0.97 (0.12 to 1.82)		1.38 (-1.41 to 5.38)		1.39 (-0.59 to 3.37)		0.41 (-2.57 to 3.39)						
HCT (L/L)	0.40 (0.37-0.44)	0.41 (0.37-0.44)	0.41 (0.37-0.46)	0.42 (0.37-0.46)	0.43 (0.40-0.47)	0.44 (0.40-0.47)	0.43 (0.37-0.48)	0.43 (0.37-0.47)	0.42 (0.38-0.46)		0.40 (0.37-0.43)				
Bias (95%CI)	0.96 (-0.04 to 1.96)		0.49 (-0.61 to 1.60)		1.41 (-1.56 to 4.38)		-1.32 (-3.03 to 0.39)		-4.56 (-6.13 to -3.00)			± 1.5	0.85	2.80	8.11
**Parameter**	**Group 1** **(N = 10)**		**Group 2** **(N = 17)**		**Group 3** **(N = 16)**		**Group 4** **(N = 7)**		**Group 5** **(N = 9)**			**APS, %**			
**Haemolysis**	**0**	**1**	**0**	**2**	**0**	**3**	**0**	**4**	**0**		**6**	**Bias**	**CV_A_**	**CV_G_**	**RCV**
MCV (fL)	87.70 (82.80-89.10)	87.60 (83.05-89.05)	88.50 (86.20-90.15)	88.50 (86.00-89.75)	90.60 (86.20-92.85)	90.40 (86.75-92.45)	89.10 (86.75-92.60)	89.00 (86.75-92.40)	90.55 (84.18-94.48)		90.05 (84.20-93.53)				
Bias (95%CI)	-0.07 (-0.43 to 0.29)		-0.31 (-0.47 to -0.15)		-0.20 (-0.60 to 0.20)		-0.21(-0.55 to 0.14)		-0.48 (-0.99 to -0.03)			± 0.9	0.38	0.80	2.45
MCH (pg)	28.30 (26.70-29.35)	28.00 (26.95-28.70)	28.50 (27.60-29.65)	28.50 (27.70-29.60)	29.30 (27.70-30.05)	29.50 (28.35-30.20)	29.70 (27.95-30.30)	30.20 (29.15-30.85)	29.05 (27.23-29.68)	30.85 (27.85-31.05)					
Bias (95%CI)	0.06 (-1.40 to 1.53)		0.16 (-0.65 to 0.98)		1.29 (-0.59 to 3.18)		2.59 (1.37 to 3.80)		4.65 (1.29 to 8.01)			± 1.35	1.01	0.80	3.57
MCHC (g/L)	322 (318-326)	320 (319-326)	322 (318-326)	324 (319-329)	324 (319-329)	327 (319-332)	324 (319-334)	334 (330-341)	321 (307-325)		332 (326-343)				
Bias (95%CI)	0.14 (-1.24 to 1.52)		0.47 (-0.4 to 1.33)		0.73 (-0.13 to 1.58)		2.81 (1.61 to 4.03)		5.24 (1.97 to 8.50)			± 0.4	1.10	1.00	4.12
RDW (%)	13.10 (11.85-15.00)	13.10 (11.95-15.10)	12.90 (12.35-13.05)	12.80 (12.40-13.10)	12.90 (12.45-14.45)	12.80 (12.45-14.25)	12.90 (12.25-13.35)	12.90 (12.25-13.35)	12.70 (12.08-14.08)	12.65 (12.23-14.15)					
Bias (95%CI)	0.63 (0.01 to 1.25)		0.12 (-0.20 to 0.45)		0.15 (-0.44 to 0.74)		0.11 (-0.43 to 0.65)		0.26 (-0.60 to 1.12)			± 1.2	0.53	1.70	4.94
Platelet count (x10^9^/L)	251 (182-252)	249 (179-292)	251 (214-275)	250 (217-274)	245 (213-284)	255 (216-295)	261 (231-302)	262 (237-303)	257 (200-301)		260 (207-319)				
Bias (95%CI)	-0.11 (-2.30 to 2.07)		-0.53 (-1.5to 0.43)		1.34 (-0.75 to 3.44)		0.63 (-2.22 to 3.49)		3.21 (0.98 to 5.44)			± 5.0	1.74	5.60	16.25
MPV (fL)	9.53 (7.48-10.75)	9.18 (7.50-10.65)	8.84 (8.08-10.16)	8.76 (8.07-10.04)	8.00 (7.62-9.64)	7.95 (7.54-9.64)	8.16 (7.19-8.63)	8.20 (7.22-8.57)	9.63 (8.32-10.73)		9.45 (8.45-10.65)				
Bias (95%CI)	-0.49 (-1.83 to 0.84)		-0.1 (-0.96 to 0.76)		-0.09 (-0.72 to 0.91)		-0.29 (-1.56 to 0.97)		-0.47 (-2.02 to 1.07)			± 1.9	0.62	2.30	6.60
Data are presented as median (interquartile range). APS - analytical performance specifications. CV_A_ - analytical variation. CV_G_ - within-subject biological variation. RCV - reference change value. WBC - white blood cell. Neu - neutrophil. Lym - lymphocyte. Mono - monocyte. Eos - eosinophil. Hb – haemoglobin. RBC - red blood cells. HCT – haematocrit. MCV - mean corpuscular volume. MCH - mean corpuscular haemoglobin. MCHC - mean corpuscular haemoglobin concentration. RDW - red cell distribution width. PLT – platelet. MPV - mean platelet volume. CI - confidence interval.															

In group 1 there was no significant difference in any of the measured parameters.

Results of group 2 showed that at HI 2 all the parameters, with the exception of MCHC, had alterations meeting the APSs. Percent bias of MCHC [0.47 (- 0.4 to 1.33)] was slightly higher than the error limit (± 0.4%) defined according to EFLM database but was still acceptable when evaluated according to RCV (4.12%).

In group 3, lymphocyte count and MCHC results had percent bias values: 7.26 (2.84 to 11.68) and 0.73 (- 0.13 to 1.58) above the APS related error limits (± 6.3 and ± 0.4, respectively), but they were acceptable in terms of RCVs (31.00% and 4.12%, respectively).

Group 4 demonstrated that for the lymphocyte count, MCH and MCHC parameters, the percent bias 7.42 (1.45 to 9.08), 2.59 (1.37 to 3.80) and 2.81 (1.61 to 4.03) were exceeding the analytical limits (± 6.3%, ± 1.35% and ± 0.4%, respectively) but all values were acceptable according to RCVs.

For the grossly haemolysed group 5, lymphocyte counts had percent bias of 11.60 (5.57 to 17.62) exceeding the analytically desirable limit (± 6.3%) but were still lower than RCV (31%). RBC count and HCT were significantly decreased in grossly haemolysed (HI 6) samples as compared with non-haemolysed ones. The percent bias results of RBC count and HCT (- 4.07 (- 5.52 to - 2.62) and - 4.56 (- 6.13 to - 3.00), respectively) were higher than the analytical bias goals (± 1.8% and ± 1.5%, respectively) but under the RCVs (7.46% and 8.11%). However, the results of MCH and MCHC in this group were affected significantly according to both of the error limits.

## Discussion

We observed that the effect of 0.11 to 7.49 g/L Hb concentration on CBC parameters were acceptable comparing to the analytical biases with the exceptions of lymphocytes, MCH, and MCHC. In addition, all the parameters were within RCV_S_. On the other hand, gross haemolysis (> 10.00 g/L Hb concentration) led to decreases in HCT, RBC count and an increase in lymphocyte count higher than the desirable bias. Moreover, variations in MCH and MCHC were exceeding the RCVs. Investigation of the impact of haemolysis on CBC is an important requirement. To our knowledge, this is the first study assessing the haemolysis interference in relation to both analytically and clinically significant error limits on the Abbott Alinity hq system.

In the present study, the effect of haemolysis on PLT count has increased in parallel to the degree of haemolysis. However, it was found to be insignificant comparing to both analytical and clinical error limits. On the other hand, De Jonge *et al.* indicated that haemolysis caused 7.3% (- 34.2% to 48.7%) and 42.6% (- 16.8% to 101.9%) bias for PLT count at mild and high degree, respectively (*11*). In another study it was reported that PLT count was biased 18.1% (4.2% to 32%) and 40.6% (20.1% to 61.1%) at mild and high degrees of haemolysis (*16*). One of the possible explanations to this discrepancy between these studies and our study may be the different analytical methodologies used. In the study by De Jonge *et al.* impedance principle was used (*11*). Platelets were counted within a specific size range based on the detection of pulses corresponding to the individual cell volume. Since the cell size was the only discriminator and both erythrocytes and PLTs were analysed in the same counting chamber of the analyser, it was suggested that non-platelet particles such as fragmented erythrocytes caused falsely increased PLT counts (*17*). In the study by Lippi *et al*., optical light scatter technique was applied for PLT enumeration (*16*). The instrument had two detectors for the measurement of low and high angle scattered light ensuring two-dimensional PLT analysis. However, the artifactual increase determined in PLT count was attributed to the interference of broken red blood cells and their cytoplasmic fragments (*18*, *19*). In the present study PLT count was also determined by an analyser using optical technique. However, this instrument performs measurements at multiple angles with additional detectors for better discrimination.

When RBC counts were evaluated, an analytically significant negative bias was observed for grossly haemolysed samples. Calculated RBC parameters like HCT, MCH and MCHC were also affected due to this decrease in RBC. These results were in compliance with the previous studies (*11*, *16*). Most remarkable variations were detected for MCH and MCHC in gross haemolysis group exceeding the allowable analytical and clinical bias.

It is reasonable to assume that cell lysis can directly affect RBC count and related indices. However, almost no significant error was determined at HI values lower than 4 (Hb concentration: 0.11-4.99 g/L) with the exception of MCHC. This was supporting the findings of De Jonge *et al.* presenting none of RBC parameter outside the acceptable limit at mild haemolysis group obtained by 5 aspirations through a fine needle (*11*). On the other hand, Lippi *et al*. found higher bias than the desirable bias for these parameters at mild haemolysis group produced by 5 aspirations corresponding to a Hb concentration between 4.0-4.5 g/L (*16*). The possible explanation to this discrepancy might be the difference in the number of times samples passed through the needle to generate mechanical haemolysis. In the present study 2, 4 and 6 times of aspirations caused samples to be haemolysed at 5 different HI levels; which probably caused by the variabilities among the fragilities of RBC membranes and their different response to trauma. According to the manufacturer’s instruction, approximate concentration ranges for HI value 1 and 2 were accepted as 0.11-1.30g/L Hb and 1.31-2.49 g/L Hb, respectively. These values were lower than corresponding concentrations expressed in study by Lippi *et al.* (*16*). Index value of 3, which corresponds to a range of 2.50-4.99 g/L Hb concentration, was relatively similar to the previous study’s mild haemolysis category. However, this range was also relatively wide and heterogeneous when compared to 4.0-4.5 g/L Hb concentration.

In the Hb analysis, RBCs were lysed using a reagent and released Hb was measured with absorption photometry. Haemoglobin measurement was not affected from haemolysis because the method was not specific for intracellular or free Hb. This finding was concordant with the previous studies (*11*, *16*).

The results of this study showed that MCV and RDW (%) were not interfered significantly with haemolysis comparing to the desirable analytical and clinical bias criteria. This finding was incompatible with the previous studies (*11*, *16*). However, these two parameters are known to be interrelated and somewhat dependent to the technology used to measure MCV. In addition, the lack of harmonization between manufacturers in volume distribution curve measurement methodologies, particularly related to exclusion of extreme values from the distribution, is suggested to result in differences between analytical systems (*20*).

Evaluation of WBC counts did not show significant bias in any of the haemolysis groups. Nevertheless, differential analysis revealed that haemolysis led to a positive bias on lymphocytes higher than desirable analytical bias in group 3, 4 and 5. On the other hand, this impact was not significant comparing with RCV.

The present study has some limitations. First, the sample size was relatively low. The study did not include HI 5 (Hb concentration: 7.50-9.99 g/L). In addition, the different reactions of the erythrocyte membranes of different samples to mechanical trauma might have been generalized with a wider study group. The second limitation of our study is that we could not find the possibility to evaluate haemolysis in pathological specimens.

In conclusion, gross haemolysis (Hb concentration > 10 g/L) is likely to produce unreliable CBC results in non-pathological samples. Further studies including pathological specimens are needed.
